# Impact of ACTH Signaling on Transcriptional Regulation of Steroidogenic Genes

**DOI:** 10.3389/fendo.2016.00024

**Published:** 2016-03-29

**Authors:** Carmen Ruggiero, Enzo Lalli

**Affiliations:** ^1^Institut de Pharmacologie Moléculaire et Cellulaire CNRS UMR 7275, Valbonne, France; ^2^Laboratoire International Associé (LIA) CNRS NEOGENEX, Valbonne, France; ^3^Université de Nice, Valbonne, France

**Keywords:** adrenal cortex, steroidogenesis, cAMP, transcription factors, gene regulation

## Abstract

The trophic peptide hormone adrenocorticotropic (ACTH) stimulates steroid hormone biosynthesis evoking both a rapid, acute response and a long-term, chronic response, via the activation of cAMP/protein kinase A (PKA) signaling. The acute response is initiated by the mobilization of cholesterol from lipid stores and its delivery to the inner mitochondrial membrane, a process that is mediated by the steroidogenic acute regulatory protein. The chronic response results in the increased coordinated transcription of genes encoding steroidogenic enzymes. ACTH binding to its cognate receptor, melanocortin 2 receptor (MC2R), stimulates adenylyl cyclase, thus inducing cAMP production, PKA activation, and phosphorylation of specific nuclear factors, which bind to target promoters and facilitate coactivator protein recruitment to direct steroidogenic gene transcription. This review provides a general view of the transcriptional control exerted by the ACTH/cAMP system on the expression of genes encoding for steroidogenic enzymes in the adrenal cortex. Special emphasis will be given to the transcription factors required to mediate ACTH-dependent transcription of steroidogenic genes.

## Introduction

The adrenal gland is a key component of the hypothalamus–pituitary–adrenal (HPA) axis, thus playing a crucial role in the adaptation of organism to stressors. The adrenocorticotropic hormone (ACTH) belongs to this regulatory circuitry, being one of the most potent physiological modulators of adrenal cortex steroidogenesis and trophicity (1, 2). It exerts its role through the binding to the G protein-coupled receptor (GPCR) melanocortin 2 receptor (MC2R), which activates adenylyl cyclase cascade leading to cAMP production and subsequent activation of cAMP-dependent protein kinase A (PKA). PKA is the main kinase responsible for the phosphorylation of specific transcription factors, which in turn regulate free cholesterol availability and activate steroidogenic enzyme expression (3, 4). Among those transcription factors, steroidogenic factor 1 (SF-1), cAMP response element-binding protein (CREB), CRE modulator (CREM), CCAAT/enhancer-binding proteins (C/EBPs), and activator protein 1 (AP-1) have been extensively described for their implication in regulating the expression of the genes encoding for steroidogenic acute regulatory (StAR) protein and steroidogenic enzymes (5–8). Any perturbation of this ACTH/cAMP/PKA-dependent cascade may cause alteration in adrenocortical cell proliferation and dysregulation of steroidogenesis as occur in various human diseases. The aim of the present review is to provide an overview of the ACTH/cAMP-dependent transcriptional regulation of the steroidogenic process in the adrenal cortex.

## Adrenal Steroidogenesis

Steroid hormones are implicated in the regulation of a plethora of developmental and physiological processes from fetal life to adulthood. Cholesterol is the precursor of all those hormones that hence share a closely related structure based on the cyclopentanophenanthrene 4-ring hydrocarbon nucleus. Cholesterol can be synthesized *de novo* from acetate in the adrenal (9). However, the main adrenal source of cholesterol is plasma low-density lipoproteins (LDLs) provided by dietary cholesterol (10). The sterol response element-binding proteins (SREBPs) are a family of transcription factors implicated in the regulation of genes participating in the biosynthesis of cholesterol and fatty acids (11). They are the main regulators of intracellular cholesterol metabolism. ACTH promotes the activation of 3-hydroxy-3-methylglutaryl coenzyme A reductase, the rate-limiting enzyme in cholesterol synthesis. It also stimulates the uptake of LDL cholesterol esters, which, once taken up by receptor-mediated endocytosis can be converted to free cholesterol for steroid hormone biosynthesis (12) or directly stored. Moreover, ACTH stimulates hormone-sensitive lipase (HSL) and inhibits acyl-coenzyme A (CoA):cholesterol acyltransferase (ACAT), thereby increasing the free cholesterol pool available for steroid hormone biosynthesis.

The initial step of steroidogenesis occurs in the mitochondria. Whereas the movement of cholesterol from the outer mitochondrial membrane (OMM) to the inner mitochondrial membrane (IMM) is known to be promoted by StAR (see below), the molecular mechanism underlying cholesterol transport and loading into the OMM is still under investigation (13). The enzymes that participate in steroid biosynthesis are either cytochrome P450s (CYPs) or hydroxysteroid dehydrogenases (HSDs).

Cytochrome P450s are a group of oxidative enzymes. In the human genome, genes for 57 CYPs enzymes have been described. Seven of them are called “type 1” enzymes. They are targeted to mitochondria and receive electrons from NADPH via a flavoprotein (ferrodoxin reductase) and a small iron-sulfur protein (ferredoxin). “Type 2” enzymes are localized in the endoplasmic reticulum and received electrons form NADPH via a single 2-flavin protein called P450 oxidoreductase (14). Six P450 enzymes participate in steroidogenesis. The mitochondrial P450 side chain cleavage (SCC, encoded by the *CYP11A1* gene) catalyzes breakage of the bond between positions 20 and 22 in the cholesterol side chain (20, 22 desmolase). P450c11β (11β-hydroxylase, encoded by the *CYP11B1* gene) and P450c11AS (aldosterone synthase, encoded by the *CYP11B2* gene) catalyze 11β-hydroxylase, 18-hydroxylase, and 18-methyl oxidase activities. At the level of the endoplasmic reticulum, we distinguish: P450c17 (encoded by the *CYP17A1* gene) that catalyzes both 17α-hydroxylase and 17,20-lyase activities; P450c21 (encoded by the *CYP21A1* gene) that catalyzes 21-hydroxylation in the synthesis of both glucocorticoids and mineralocorticoids; and P450-Aro (encoded by the *CYP19A1* gene) that catalyzes the aromatization of androgens to estrogens.

The HSDs use nicotinamide adenine dinucleotides or their phosphate forms (NADH/NAD^+^ or NADPH/NADP^+^) as cofactors to either reduce or oxidize a steroid through a hydride transfer mechanism (15). Differently from steroidogenic reactions catalyzed by P450 enzymes, which are mediated by a single form of P450, each reaction catalyzed by HSDs involves at least two, often different isozymes. On the basis of their structures, those enzymes are divided into: short-chain dehydrogenase/reductase (SDR) family, which include 11β-HSDs 1, 2, and 17β-HSDs 1, 2, and 3 and aldo-keto reductase (AKR) family, which include 17β-HSD5 (15, 16). Under a physiological point of view, it is preferable to classify the HSDs as dehydrogenases or reductases. The former use NAD^+^ as cofactors to oxidize hydroxysteroids to ketosteroids, whereas the latter mainly use NADPH to reduce ketosteroids to hydroxysteroids. Those enzymes act *in vitro* typically bidirectionally on the basis of the pH and cofactor concentration, while in intact cells they work mainly in one direction, with the direction established by the cofactors available (15, 16).

The pattern of steroid hormones secreted by each adrenal zone is determined by the enzymes expressed in each zone (17). Specifically, adrenal *zona glomerulosa* expresses angiotensin II receptors and P450c11AS, whereas it does not express P450c17. Indeed, *zona glomerulosa* produces aldosterone under the regulation of the renin–angiotensin system. In contrast, at the levels of *zona fasciculata*, angiotensin II receptors, and P450c11AS are not detected, whereas the ACTH receptor MC2R and P450c11β are expressed (18). The *zona fasciculata* also expresses P450c17, which catalyzes 17α-hydroxylation, exhibiting only little 17,20-lyase activity. Indeed, *zona fasciculata* secretes the two glucocorticoids, cortisol and corticosterone, under the influence of ACTH, but very little dehydroepiandrosterone (DHEA). Interestingly, patients displaying mutations in P450c17 are not able to produce cortisol, but show increased corticosterone production (19), like in rodents, which normally do not express P450c17 in their adrenals. Regarding the *zona reticularis*, it also expresses MC2R and large amounts of P450c17 and cytochrome b_5_, thus displaying 17,20-lyase activity with subsequent DHEA production, the most part of which is sulfated to DHEAS by SULT2A1. In general, small amounts of DHEA are converted to androstenedione, and very little amounts of androstenedione are converted to testosterone, likely through AKR1C3/17βHSD5. In contrast, *zona reticularis* expresses very little P450c21 and P450c11β (thus producing only a minimal amount of cortisol) and relatively little 3βHSD2.

## Acute Response to ACTH: The Steroidogenic Acute Regulatory Protein

Steroidogenic cells are able to store very little amounts of steroids, which imply a rapid synthesis of new steroids in response to a sudden demand. ACTH exerts its role in promoting steroidogenic cell growth and stimulating steroidogenesis at three distinct levels. We first distinguish a long-term exposure to ACTH. It takes weeks or months to stimulate adrenal growth and it is mediated by ACTH-dependent production of cAMP, which in turn triggers IGF-II (20), fibroblast growth factor (FGF) (21), and epidermal growth factor (EGF) (22) synthesis. The concerted action of those growth factors is to promote adrenal cellular hypertrophy and hyperplasia. Second, ACTH can act over days through cAMP to stimulate the transcription of genes, which encode for different steroidogenic enzymes (see below). Third, ACTH can mediate an acute response, which occurs within minutes and is inhibited by protein synthesis inhibitors (like puromycin or cycloheximide). This ACTH-mediated acute response is accompanied by a rapid stimulation of the StAR gene transcription in steroidogenic cells of the adrenal cortex, testis, and ovary (5, 23) and by the phosphorylation of Ser195 in the existent pool of StAR (24). Those events promote cholesterol flow from the OMM to the IMM, where cholesterol is converted to pregnenolone in the first and rate limiting step of steroid hormone biosynthesis. The first to show that ACTH acute steroidogenic response involved the rapid synthesis of a 37-kDa phosphoprotein were Orme-Johnson and coworkers (25, 26). Just a few years later, Stocco and colleagues cloned this protein and they gave it the name of “StAR” (27). StAR is an acutely regulated, cycloheximide-sensitive protein exhibiting a mitochondrial leader sequence by which it is directed to the mitochondria. Once inside the mitochondria, StAR is cleaved to a 30-kDa protein. It has been shown that the overexpression of a mouse StAR in the mouse Leydig MA-10 cells increased their basal steroidogenesis (27). Moreover, when expression vectors for both StAR and P450scc enzyme are cotransfected in non-steroidogenic COS-1 cells, the synthesis of pregnenolone is augmented respect to that obtained with P450scc alone (28). The pivotal role of StAR in the regulation of steroidogenesis was strengthened after the identification of mutations causing premature stop codons in the StAR gene of patients affected by the most common form of congenital lipoid adrenal hyperplasia (CAH) (28, 29), a rare disorder of steroid biosynthesis. In this disorder, glucocorticoid, mineralocorticoid, and sex steroids biosynthesis is impaired, which may lead to adrenal failure, severe salt wasting crisis and hyperpigmentation in phenotypical female infants irrespective of genetic sex (29). Moreover, it has been shown that in mice the targeted disruption of the *Star* gene causes defects in steroidogenesis, with consequent male pseudohermaphroditism and lethality within 1 week after birth (30, 31).

Several studies have been carried out to understand the mechanism of action of StAR (32), which still remains to be fully elucidated. It was hypothesized that the “mature” 30-kDa intramitochondrial form of StAR was the biologically active portion of the protein, due to its longer half-life respect to the short one of the 37 kDa precursor. However, when the two StAR forms are expressed in the cytoplasm or added to mitochondria *in vitro*, they are equally active (33). Moreover, while constitutively active when it is localized on the OMM, StAR results to be inactive at the level of the mitochondrial intramembranous space or the matrix (34). That evidence suggested that StAR exerts its action on the OMM, its steroidogenesis-promoting function tightly depending on the residency time on the OMM itself (33, 34). This implies that StAR activity is linked to its localization rather than to its cleavage to the “mature” form. When StAR interacts with the OMM, it undergoes to conformational changes (35, 36) that allow StAR to accept and discharge cholesterol molecules. Interestingly, steroidogenesis-promoting and cholesterol-transfer activities of StAR are distinct. Indeed, StAR-mediated transfer of cholesterol between synthetic membranes *in vitro* (37) is maintained also by the inactive R182L mutant, which impairs steroidogenesis, causing lipoid CAH (38). Finally, StAR activity on the OMM requires the translocator protein TSPO, also called peripheral benzodiazepine receptor (PBR) (39, 40), which has been identified as a key player in the flow of cholesterol into mitochondria to permit the initiation of steroid hormone synthesis. Moreover, it has been demonstrated that phosphorylated StAR interacts with voltage-dependent anion channel 1 (VDAC1) on the OMM, which in turn promotes processing of the 37-kDa phospho-StAR to the 32-kDa intermediate (41). In the absence of VDAC1, phospho-StAR undergoes degradation by cysteine proteases prior to mitochondrial import and subsequent cleavage to the 30-kDa protein. StAR phosphorylation by PKA requires phosphate carrier protein on the OMM, which seems to interact with StAR before it interacts with VDAC1 (41). Importantly, although StAR is necessary for the ACTH-mediated acute steroidogenic response, steroidogenesis still occurs in the absence of StAR (around 14% of StAR-induced rate) (29, 42). This may account for the steroidogenic capacity of tissues lacking StAR, like placenta and brain.

## Chronic Response to ACTH

**Figure 1:**
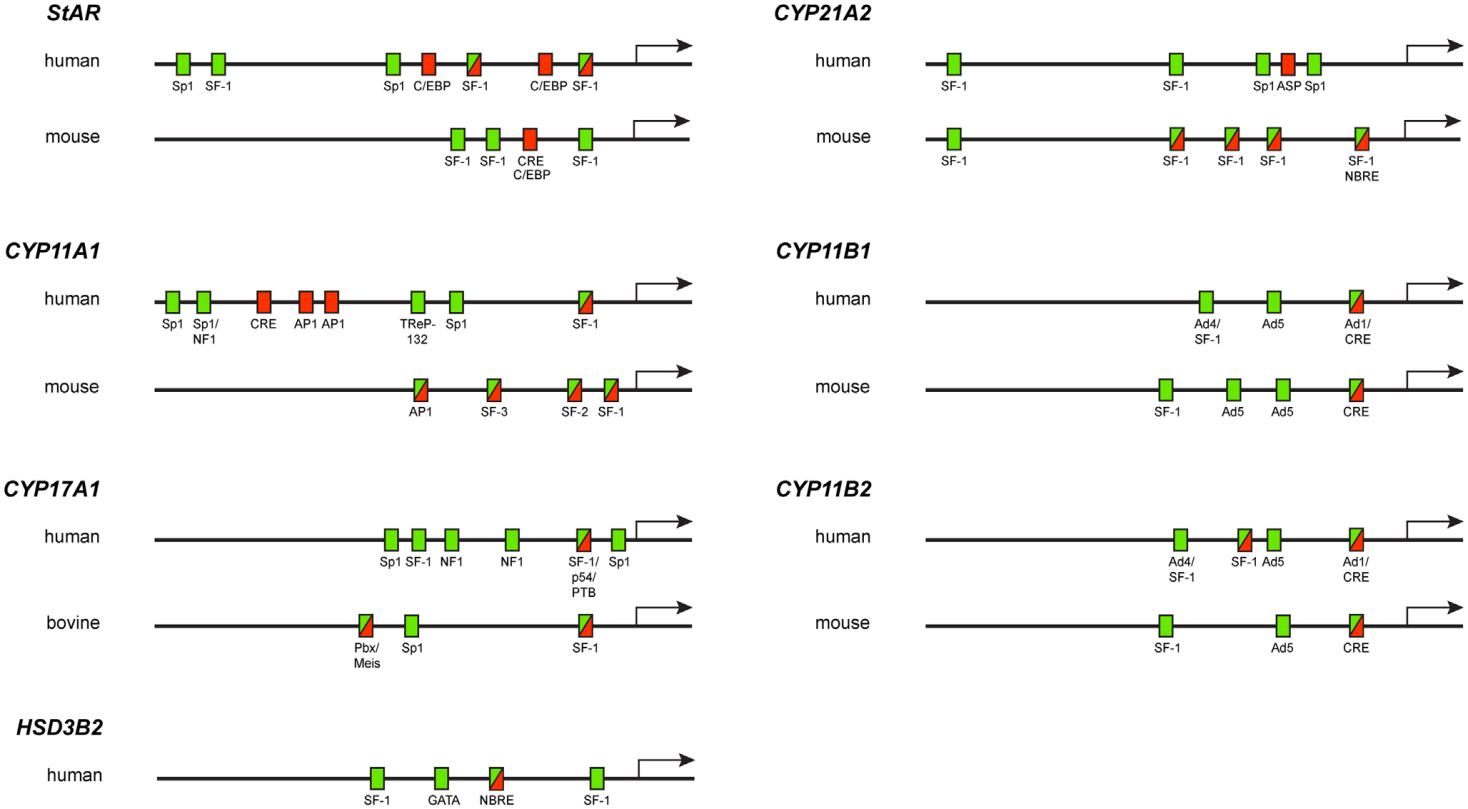
**Diagram showing the localization of elements required for basal activity (green rectangles), cAMP-regulated activity (red rectangles) or both basal and cAMP-regulated activities (green–red rectangles) in steroidogenic gene promoters**.

The transcription of steroidogenic genes depends on the slower, chronic response to ACTH in the adrenal cortex. Indeed, ACTH interaction with specific membrane receptors leads to the activation of coupled G proteins, with subsequent stimulation of membrane-associated adenylyl cyclase catalyzing cAMP formation. cAMP-activated PKA hence phosphorylates multiple transcription factors, whose concerted action and interaction with different *cis*-regulatory elements direct StAR and steroidogenic gene expression. Furthermore, after transcription factor binding to gene promoters, posttranslational modifications, like phosphorylation/dephosphorylation and coactivator proteins binding, are required to activate gene expression. In the following section, the main transcription factors that direct the transcription of steroidogenic genes in response to ACTH will be described (Table 1; Figure 1).

**Table 1:** **Promoter elements implicated in basal and ACTH/cAMP-regulated expression of steroidogenic genes**.

	Basal regulation	ACTH/cAMP-dependent regulation
**StAR**	Three SF-1-binding sites [−135; −95; −45; 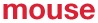 promoter, Ref. (7)] Three SF-1-binding sites [−926/−918; −105/−96; −43/−36; 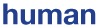 promoter, Ref. (43, 44)] Two Sp1-binding sites [−1159/−1153; −157/−151; 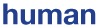 promoter, Ref. (44, 45)]	CRE2/AP-1 [−81/−75, 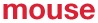 promoter; Ref. (6)] Two C/EBPs putative-binding sites [−119/−100; −50/−41; 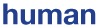 promoter, Ref. (43)] Two SF-1-binding sites [−105/−65; −43/−36; 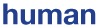 promoter, Ref. (43)] Highly conserved overlapping motif [−81/−72, 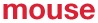 promoter, which recognizes the CRE/AP1 and C/EBPs family of proteins, Ref. (7, 46–48)]
**CYP11A1**	Proximal SF-1-binding site [P site, −46/−38; 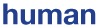 promoter, reviewed in Ref. (49)] Imperfect Sp1-binding site [−111/−101; 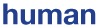 promoter, reviewed in Ref. (49)] TReP-132 [−155/−131; 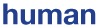 promoter, reviewed in Ref. (49)] Adrenal enhancer (AdE, −1850) composed of two binding regions (a) AdE1 (−1845) containing an imperfect Sp1 and an NF-1-binding site [ 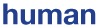 promoter, reviewed in Ref. (49)] (b) AdE2 (−1898) containing an imperfect Sp1-binding site [ 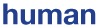 promoter, reviewed in Ref. (49)] AP-1 motif [−319/−313; 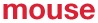 promoter, Ref. (50)] TGAGTCA motif [termed SF-3-binding site, −120/−114; 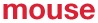 promoter, Ref. (50, 51)] AGGTCA motif [termed SF-2-binding site, −73/−68; 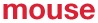 promoter, Ref. (50, 51)] AGCCTTG motif [termed SF-1-binding site, −45/−39; 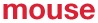 promoter, Ref. (50, 51)]	Proximal SF-1-binding site [P site, −46/−38; 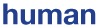 promoter, reviewed in Ref. (49)] Upstream cAMP responsive sequence (U-CRS, −1600 bp), which includes (a) SF-1-binding site (−1617/−1609) (b) CREB/ATF-binding site (CRE; −1685/−1606) (c) Two AP-1-binding sites (−1559/−1553; −1633/−1626) (a)/(b)/(c) 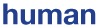 promoter, reviewed in Ref. (49) AP-1 motif [−319/−313; 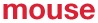 promoter, Ref. (50), although available data indicate that this motif is not a major contributor to the induction of CYP11A1 expression by ACTH/cAMP] TGAGTCA motif [termed SF-3-binding site, −120/−114; 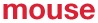 promoter, Ref. (50, 51)] AGGTCA motif [termed SF-2-binding site, −73/−68; 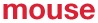 promoter, Ref. (50, 51)] AGCCTTG motif [termed SF-1-binding site, −45/−39; 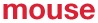 promoter, Ref. (50, 51)] (mutation of those elements reduced the expression levels of *Cyp11A1* gene following treatment with 8-Br-cAMP, although all mutated plasmids retained appreciable responsiveness to cAMP)
**CYP11B1**	CRE-binding site [termed Ad1/CRE and resembling a consensus CRE, −71/−64; 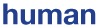 promoter, Ref. (52, 53) reviewed in Ref. (54)] Ad5 [−119/−111; 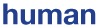 promoter, ERRalpha has been shown to be the nuclear protein interacting with this element under basal conditions, reviewed in Ref. (54)] SF-1-binding site [termed Ad4, −242/−234; 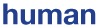 promoter, it seems to be less important for both CYP11B1 and CYP11B2 basal expression, reviewed in Ref. (54)] CRE-binding site [−74/−67; 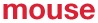 promoter, reviewed in Ref. (54)] Two Ad5-binding sites [one at −122/−114 and the other one at −208/−200; 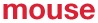 promoter, reviewed in Ref. (54)] SF-1-binding site [−247/−240; 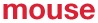 promoter, reviewed in Ref. (54)]	CRE-binding site [termed Ad1/CRE and resembling a consensus CRE, −71/−64; 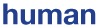 promoter, Ref. (52, 53), reviewed in Ref. (54)] Functional CRE consensus sequence [−56/−49, 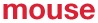 promoter, Ref. (55)]
**CYP11B2**	CRE-binding site [termed Ad1/CRE and resembling a consensus CRE, −71/−64; 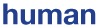 promoter; Ref. (53), reviewed in Ref. (54)] Ad5 [−119/−111; 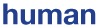 promoter, ERRalpha has been shown to be the nuclear protein interacting with this element under the basal conditions, reviewed in Ref. (54)] Two SF-1-binding sites [one at −129/−114, 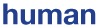 promoter, Ref. (56)]; The other one termed Ad4, −344/−337, 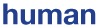 promoter. It seems to be less important for both CYP11B1 and CYP11B2 basal expression; reviewed in Ref. (54) Chicken ovalbumin upstream promoter transcription factor [COUP-TF, −129/−114; 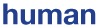 promoter, Ref. (56)] CRE-binding site [−67/−60; 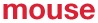 promoter, reviewed in Ref. (54)] Ad5 [−108/−100; 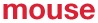 promoter, reviewed in Ref. (54)] SF-1 [−330/−323; 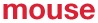 promoter, reviewed in Ref. (54)]	CRE-binding site [termed Ad1/CRE and resembling a consensus CRE, −71/−64; 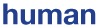 promoter; Ref. (53, 56) reviewed in Ref. (54)] SF-1-binding site [−129/−114; 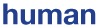 promoter, Ref. (56)] Chicken ovalbumin upstream promoter transcription factor [COUP-TF, −129/−114; 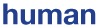 promoter, Ref. (56)] CRE-binding site [−67/−60; 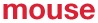 promoter, Ref. (56), reviewed in Ref. (54)]
**CYP17A1**	ASP/Sp1-binding site [−8/−19; 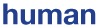 promoter, Ref. (57)] SF-1-binding site [−58/−50; 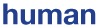 promoter, Ref. (57)] Two nuclear factor 1 (NF-1)-binding sites [−107/−85; −178/−152, 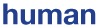 promoter, Ref. (59)] −184/−206 region [The site within this sequence that confers basal activity is not known, although it does contain a sequence resembling an SF-1 site at −195/−200; 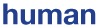 promoter, Ref. (57)] Sp1/Sp3-binding site [−227/−184, 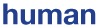 promoter; Ref. (59)] SF-1 (−62/−40), Sp1 (−186/−177), and Pbx/Meis (−243/−225) binding sites [ 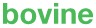 promoter; Ref. (59–63)]	cAMP-regulatory sequence [CRS, −57/−38: SF-1, p54nrb/NonO, and poly-pyrimidine tract-binding protein-associated splicing factor (PSF) are the transcription factors shown to be associated to this region; 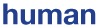 promoter, Ref. (58)] SF-1 (−62/−40)- and Pbx/Meis (−243/−225)-binding sites [ 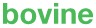 promoter; Ref. (60–63)]
**CYP21**	Two Sp1-binding sites [−118/−112 and −106/−100 within the recognition site −129/−96; 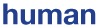 promoter, Ref. (64)] Two SF-1-binding sites [a putative one within the −300 bp proximal promoter and a second one within a distal region, lying approximately 4.8 kb upstream of the transcription start site; 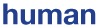 promoter, Ref. (65)] Enhancer element [−330/−150; 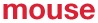 promoter, Ref. (51)] Essential regulatory element [−210/−170; 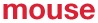 promoter, highly conserved in the genes from human and bovine; Ref. (51, 66, 67)] A and B elements located 5.3 and 6 kb upstream of the transcriptional start site [ 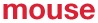 promoter, Ref. (69)]	Adrenal-specific protein (ASP)-binding site [−129/−113, within the recognition site −129/−96; 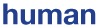 promoter, Ref. (64)] Enhancer element [−330/−150; 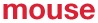 promoter, Ref. (51)] Essential regulatory element [−210/−170; 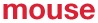 promoter, highly conserved in the genes from human and bovine; Ref. (51, 66, 67)] cAMP consensus sequence [−68/−62; 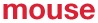 promoter, it matches part of the consensus sequence proposed for cAMP-regulated expression of other genes, Ref. (68)] Nuclear-binding response element (NBRE)/Nurr77 binding site [−65, 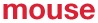 promoter, Ref. (66)] Regulatory elements containing variation of an AGGTCA motif at −170, −210, −140, −65 [ 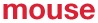 promoter; they show similarity to the CRE consensus, although they do not function as classical CREs, Ref. (68); variations of these same AGGTCA-bearing elements are also involved in the expression of *Cyp11a* and *Cyp11b* in Y1 adrenocortical cells, see above and Ref. (70)]
**HSD3B2**	Two SF-1/LRH-1-binding sites [−64/−56; −315/−307; 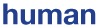 promoter, reviewed in Ref. (71)] Nuclear-binding response element (NBRE)/Nurr77 binding site [−131; 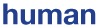 promoter, reviewed in Ref. (71)] GATA-binding site [−196/−190; 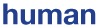 promoter, reviewed in Ref. (71)]	Nuclear-binding response element (NBRE)/Nurr77 binding site [−131, 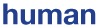 promoter, reviewed in Ref. (71)]

*Blue, red and green colours distinguish the different species (human, mouse and bovine, respectively)*.

### Steroidogenic Factor 1

Steroidogenic factor-1 (SF-1; NR5A1) is an orphan member of the nuclear receptor superfamily, which acts as a key regulator of adrenogonadal development and tissue-specific gene expression in steroidogenic cells. Parker and Morohashi groups identified SF-1 by its capacity to bind to and activate transcription from multiple P450 steroidogenic enzyme promoters, which display one or more SF-1-binding sequences in close proximity to the TATA box (72, 73). Furthermore, it has been shown that SF-1 interacts with multiple coactivator and corepressor proteins that function as bridges between transcription factors and the basal transcription system (74–76).

In the human adrenocortical cancer cell line H295R *StAR* gene transcription is induced by both angiotensin II and cAMP via increased SF-1-binding to a cAMP-responsive region within the first 350 bp upstream of the transcription initiation site (45, 77–79). On the other hand, the mutation of the SF-1 response elements in the mouse promoter of the StAR gene does not impair cAMP-dependent StAR induction in MA-10 Leydig cells or Y1 adrenocortical cells (78).

Adrenocorticotropic hormone/cAMP-dependent transcription of the human *CYP11A1* gene, encoding for the mitochondrial enzyme P450scc responsible for cholesterol conversion to pregnenolone, requires the binding of SF-1 at two sites (−40 and −1600 bp) on the gene promoter (80). Remarkably, Omura and Chung laboratories have shown that the upstream promoter elements confer a large portion of CYP11A1 responsiveness to cAMP in Y1 or I-10 Leydig tumor cells and primary adrenal cells of transgenic mice (80, 81). SF-1-dependent activation of the *CYP11A1* promoter can be potentiated by cotransfection with c-Jun in steroidogenic JEG3 cells, but not in COS-1 cells (82). Thus, c-Jun and SF-1 act synergistically to activate the transcription of *CYP11A1*. It has also been shown that in the human adrenal cortex *CYP11A1* can be strongly activated by GATA-6 in a SF-1-dependent and DAX1-sensitive fashion (83).

Moreover, it has been shown that SF-1 interacts with the homeodomain-containing transcription factor pituitary homeobox 1 (Ptx/Pitx1) to synergistically promote *CYP11B1* gene transcription (84). Interestingly, in the bovine adrenal only one *CYP11B1* gene is expressed. Its promoter is characterized by the presence of both a SF-1- and a CRE (cAMP-response element)-like binding site, which are essential for cAMP-driven transcription (85, 86).

Adrenocorticotropic hormone/cAMP signaling also regulates the expression of the human *CYP11B2* gene in the *zona glomerulosa*, where it is responsible for mineralocorticoid production via an SF-1-binding site and a CRE (56).

Furthermore, SF-1 is implicated in the transcriptional regulation of *CYP17A1*, the gene encoding the P450c17 enzyme, which catalyzes both the 17α-hydroxylation of pregnenolone and progesterone (required for cortisol biosynthesis) and the 17,20-lyase reaction of 17α-hydroxylated steroids (leading to androgen production). Studies carried out on H295R cells revealed that CREs are located within the first 63 bp upstream of the transcriptional initiation site and that a second basal transcription element lies between −184 and −206 bp (87). SF-1 forms a complex with p54^nrb^/NonO and polypyrimidine tract-binding protein-associated splicing factor (PSF), which binds within those first 60-bp upstream of the transcriptional start site, stimulating CYP17 expression in response to ACTH/cAMP signaling (58). As for *CYP11A1*, GATA-6 promotes the SF-1-dependent transcription of *CYP17* in H295R cells (83).

Many studies have revealed that the ACTH-regulated expression of the *CYP21A1* gene, coding for the P450c21 enzyme, which has a key role in the production of cortisol and aldosterone, requires the binding of the nuclear receptors SF-1 and Nur77 to its promoter (88–90). Interestingly, SF-1 binds to a distal region that lies approximately 4.8 kb upstream of the *CYP21A1* transcription start site driving adrenal-specific expression of the human gene (65).

### cAMP Response Element/Binding Protein/CRE Modulator/Activating Transcription Factor

A family of cAMP-responsive nuclear factors mediates transcriptional regulation by ACTH/cAMP signaling pathway. This family is composed by a large number of proteins, which are encoded by the CREB, CREM, and ATF genes. Those proteins recognize and bind the 8-bp 5′-TGACGTCA-3′ palindromic sequence or a minor variation, called the CRE, which lie within 100 nucleotides of the TATA box in the promoters of eukaryotic cAMP responsive genes (91–93). The members of the CREB family are characterized by their DNA-binding leucine zipper (bZIP) domains and generally they interact with each other to mediate cAMP-dependent transcriptional response (91). Interestingly, the sequences of the mouse (27), human (94), and rat *StAR* promoters, which exhibit an extensive homology, lack a consensus CRE, similarly to the promoters of different steroid hydroxylase genes whose transcription is regulated by ACTH/cAMP signaling (57). It has been shown that the cAMP-responsive region of the StAR gene promoter exhibits a highly conserved motif (50-TGACTGATGA-30 corresponding to 281/272 bp in the mouse promoter) to which different bZIP families of transcription factors, like not only CREB, CREM, and ATF1, but also AP-1 and C/EBPs (see below) bind to drive StAR transcription (7, 46, 47, 95, 96).

cAMP response element-binding protein has been demonstrated to be the principal player in mediating stimulus-transcription coupling in the ACTH/cAMP pathway. However, knockout mouse CREB studies showed that this action can be compensated by other CRE-binding proteins like CREM and ATF-1 (97). This mechanism also seems to work in the regulation of steroiodogenesis, as CREB family members directly induce *StAR* gene transcription (6, 7, 47). Interestingly, whereas CREB gene products usually function as positive transactivators, CREM can either activate or repress CRE-mediated transcription (98, 99). Alternative splicing of the CREM gene originates multiple isoforms that can act as either activators (τ, τ1, and τ2) or repressors (α, β, and γ) of transcription (100). Identical functional regions have been identified in CREB and CREMτ proteins (99). When overexpressed, either CREB or CREMτ display qualitatively comparable effects toward cAMP-dependent StAR gene transcription in murine adrenal and gonadal cells (6, 7, 101), whereas CREMα and CREMβ have been shown to repress StAR transcription (7). Further, CREM proteins can bind to CREs as homodimers or as heterodimers with CREB/ATF displaying similar functional outcomes to those of CREB (91). Remarkably, it has been shown that CREB and CREM associate with the proximal rather than the distal StAR promoter upon cAMP analog treatment (47, 48). Sugawara and coworkers compared the implication of CREB and CREM in cAMP-mediated StAR gene expression and identified CREM as the main mediator in H295R cells (101). In contrast, another group showed that CREB and ATF-1, but not CREM, mainly bound to the StAR promoter upon ACTH/cAMP stimulation (47). Besides CREB and CREM, also the CRE-binding protein, ATF-1 is a key regulator of StAR gene expression. ATF-1 differs from CREB and CREM as it lacks the glutamine-rich Q1 domain, although this does not affect its ability to work as a transcriptional activator (91, 102). Interestingly, two paralogs of ATF-1, called CRE-binding protein 1(CRE-BP1 or ATF-2) and ATF-a, display alternative exon splicing and bind to CREs, but they are not able to mediate cAMP-responsive transactivations (91).

cAMP response element-binding protein/CREM/ATF are activated by PKA, PKC, and other kinases that phosphorylate them at specific residues within their N-terminus. Indeed, phosphorylation of CREB at Ser133/119 or CREM at Ser117 leads to CREB–CREM interaction with coactivators like CREB-binding protein/p300 (CBP/p300) (see below) with subsequent stimulation of their transcriptional activity (47, 103–105). It has been reported that cAMP analogs increase CREB phosphorylation in a time-dependent manner in steroidogenic cells. This phosphorylation event correlates with the association of both phosphorylated CREB and CBP to the proximal promoter of the StAR gene (47, 48, 106). The phosphorylation of Ser133 is required for CREB activation; however, it is not sufficient for full activation of the protein. Indeed, it has been shown that a short region C-terminal to the PKA phosphorylation site within CREB protein is required for CREB transcriptional activation (91, 107, 108). The crucial role of CREB phosphorylation by PKA has been also observed *in vivo* where transgenic mice, which express a non-phosphorylatable mutant of CREB (called CREB-M1, Ser133Ala), exhibit somatotroph hypoplasia and dwarfism (109). Moreover, expression of CREB-M1 in adrenal and gonadal cells strongly decreases cAMP-induced StAR gene expression (6, 7, 101).

cAMP response element-binding protein participates together with GATA-6 and AP-1 to the SF-1- and AP-2-dependent *CYP11A1* gene transcription in rodent placenta and ovary (110). Interestingly, the ACTH-stimulated transcription of the human *CYP11B1* gene depends on the CREB family member ATF-2 (52). Moreover, as reported above, ACTH/cAMP signaling regulates *CYP11B2* gene transcription via a CRE and a SF-1-binding site (56). Similarly, CREB binds to a CRE at the level of the mouse *Cyp11b1* gene promoter to drive ACTH-dependent transcription (55).

### CCAT/Enhancer-Binding Proteins

CCAT/enhancer-binding proteins are a family of transcription factors containing a highly conserved bZIP at the C-terminus that is involved in dimerization and DNA binding. C/EBPs bind with different affinities to a consensus site consisting of a dyad symmetrical repeat (A/GTTGCGC/TAAC/T) (111). At least six members of the family have been cloned and characterized, named from C/EBPα to C/EBPζ. C/EBPα and C/EBPβ are expressed in steroidogenic cells, the expression levels of C/EBPβ being increased in the nucleus by the action of LH and analogs of cAMP (112, 113). The cAMP-inducible domains of C/EBPs, with the exception for C/EBPβ, lack a PKA phosphorylation site, which implies that C/EBPs are able to mediate cAMP-dependent responses by indirect mechanisms. In contrast, C/EBPβ phosphorylation by PKA within its bZIP domain affects its DNA-binding activity (114).

C/EBPβ targets a binding region in the promoter of the mouse *Star* gene (281/272 bp), which is also bound by CREB/ATF (see above) and Fos/Jun (see below). Further, two putative C/EBP-binding sites have been identified within the human StAR promoter (43). Consequently, an implication for both C/EBPα and C/EBPβ in StAR gene transcription has been reported (43, 115, 116). C/EBPβ phosphorylation on Thr325 increases its association to the proximal StAR promoter, thus inducing StAR transcription (117, 118). Interestingly, it has been shown that GATA-4 and C/EBPβ directly interact *in vitro* and *in vivo* and synergistically activate the StAR promoter only in the presence of PKA (95, 117). This suggests that GATA-C/EBP transcriptional cooperation might promote ACTH/cAMP-dependent StAR transcription in all steroidogenic tissues, as this kind of PKA-dependent synergy has been shown for other members of GATA and C/EBP families. Finally, the disruption of either C/EBPα or C/EBPβ impairs normal reproductive development in female mice, with consequent reduced or altered ovulation and corpus luteum formation inability (112, 119).

### AP-1 Family of Transcription Factors

The AP-1 family of transcription factors participates in the regulation of cellular responses to multiple stimuli regulating proliferation, transformation, and cell death (120). It recognizes and binds to a DNA sequence known as the AP-1/phorbol 12-*O*-tetradecanoate 13-acetate responsive element [AP-1/TRE; TGA(C/G)TCA]. AP-1 is composed by a mixture of homo- and hetero-dimers formed between Jun (c-Jun, JunB, and JunD) and Fos (c-Fos, FosB, Fra1, and Fra2) family members (121–123). Fos members heterodimerize with Jun proteins and with specific members of the CREB/ATF family, but they are not able to form homodimers, whereas Jun members function as homodimers or heterodimers among themselves or with members of the Fos and CREB/ATF families (124). Both Jun and Fos family proteins belong to the bZIP group of DNA-binding transcription factors and their dimerization is necessary for specific and high affinity binding to the palindromic DNA sequence TGAC/GTCA (see above) (125). Studies carried out on the mouse *Star* promoter identified a highly conserved element (TGACTGA, −81/−75 bp), which shows homology with the AP-1/TRE sequence and overlaps also with the CRE2 sequence (6). Interestingly, it has been shown that Fos and Jun bind to this element, called CRE2/AP-1, thus regulating *Star* gene transcription (46, 96, 126). Moreover, two additional putative AP-1-binding sites have been identified within the rat *Star* promoter. c-Fos reduces basal, cAMP-, and c-Jun-mediated rat *Star* gene transcription in Y-1 adrenocortical cells (127). Indeed, a functional comparison between Fos and Jun revealed that c-Jun is the most powerful AP-1 family member for *Star* gene transactivation. Accordingly to this, it has been shown that only c-Jun, but not other AP-1 members, plays a pivotal role in the regulation of PKC-mediated *Star* transcription and steroidogenesis in Leydig and adrenal cells (96, 128).

Not only Protein kinase A but also PKC phosphorylate several Ser and Thr residues on c-Jun and c-Fos. In particular, the treatment with a cAMP analog or a growth factor increases the phosphorylation of c-Jun Ser63 and c-Fos Thr325. Those phosphorylation events are associated to StAR gene transcription and steroidogenesis in mouse Leydig cells (47, 96, 126, 129). Interestingly, ACTH/cAMP-dependent c-Jun and c-Fos phosphorylation increases the association between p-c-Jun/p-c-Fos and the CBP/p300 cofactors (see below), with consequent recruitment of CBP/p300 to the StAR promoter (47, 96, 126). The phosphorylation of c-Fos and c-Jun can alter their capacity to interact with other transcription factors, affecting their dimerization and DNA-binding specificity (124). This explains why the crosstalk between CREB and c-Fos/c-Jun can be associated with both gain and loss of function on the same *cis*-element in the context of a fine regulation of the transregulatory elements which participate in StAR gene transcription (126).

### Sp Family of Transcription Factors

The Sp family of transcription factors is characterized by the presence of three conserved Cys2His2-type zinc fingers at their C-terminus that form the sequence-specific DNA-binding domain (130). They can bind and exert their action through GC/GT-rich promoter elements to regulate the expression of multiple target genes (130, 131). Sp1 is the most well characterized member of the family and it exhibits similar structure and a high homology with Sp3 at the level of its DNA-binding domains, both being able to enhance ore repress promoter activity. However, although Sp1 and Sp3 recognize the same consensus-binding sites, it has been reported that both their DNA-binding properties and regulatory functions differ and depend on the promoter context and cellular background (130, 132, 133).

Regulatory elements for Sp1 and Sp3 have been identified within the human TSPO proximal promoter (134). They have been demonstrated to be strong positive elements for the promoter activity, although differences have been detected in the mechanism by which TSPO is regulated in non-steroidogenic versus steroidogenic cells (134). Sp1 is also involved in the cAMP-dependent transcription of the *CYP11A1* gene in human (135), bovine (136, 137), and porcine (138) adrenals. Furthermore, a cooperation between SF-1, Sp1, and CBP has been reported to drive cAMP-dependent *CYP11A1* transcription in bovine adrenal (136). Sp1 and Sp3, together with the nuclear fator-1C (NF-1C), bind to the second basal element of the *CYP17* gene promoter. This event is crucial for optimal basal transcription (59). Sp1 can also form a complex with GATA-4 or GATA-6 to regulate the expression of *CYP17* in the adrenal gland (139). Finally, Sp1 and adrenal-specific protein (ASP) bind to the *CYP21* gene promoter to regulate its cAMP-dependent transcription (140). This synergistic action seems to be required for maximal *CYP21* induction (140).

### DAX-1

*DAX-1/NR0B1* encodes an unusual member of the nuclear hormone receptor family of transcription factors. Its mutations cause adrenal hypoplasia congenita (AHC) associated with hypogonadotropic hypogonadism (HHG) (141, 142). *DAX-1* expression pattern, mostly restricted to steroidogenic tissues, suggested that it may have a role in the regulation of steroidogenesis. Indeed, in adrenocortical cells, DAX-1 works as a global negative regulator of basal and cAMP-regulated transcription of steroidogenic genes, both through direct binding to gene promoters and interaction with SF-1 and other transcription factors [(143–145); reviewed in Ref. (146)]. In addition, consistently with DAX-1 negative action on steroidogenesis, activation of the PKA pathway by ACTH in adrenocortical cells (147) and FSH in Sertoli cells (148) downregulates Dax-1 expression.

## Role of Coregulators

Transcriptional coregulators are crucially implicated in nuclear receptor-mediated transcriptional activation (149–151) and transactivation by other factors, exerting their roles in multiples processes, like histone modification (152–154), chromatin remodeling (155), post-translational modification of transactivation complex members (156, 157), and ordered recruitment of basal transcriptional machinery (158, 159).

The role of coactivators and corepressors in the transcriptional regulation of steroid hydroxylase genes and *StAR* has been shown by several studies (74, 76). CBP and its functional homolog p300 are transcriptional coactivators that contain multiple functional domains and display intrinsic histone acetyltransferase activity (151, 160), by which they increase transcription factor accessibility to nucleosomal DNA. Classically, ACTH/cAMP signaling triggers the phosphorylation of specific transcription factors, which in turn can bind and recruit CBP/p300 (105, 160–162). As already reported (see above), when phosphorylated at Ser133 CREB is able to interact with CBP (104, 105). Interestingly, CBP/p300 contain PKA consensus sites, the phosphorylation of which is involved in regulating their functions. CBP/p300 play a key role in the transcriptional regulation of the *StAR* gene. Different studies have shown that cAMP-dependent phosphorylation of CREB at Ser133, c-Jun at Ser63, and c-Fos at Thr325 promotes the association and recruitment of CBP/p300 to the proximal *StAR* promoter (47, 48, 95, 126). cAMP-stimulated phosphorylation of C/EBPβ at Thr325 also increases C/EBPβ association to the proximal *StAR* promoter (117). Other factors like SF-1 and GATA-4 (see above), that are bound to the proximal *StAR* promoter, once phosphorylated in response to ACTH might enhance CBP/p300 recruitment to the promoter. This correlates with the CBP/p300-dependent increased effects of C/EBPβ and GATA-4 on *StAR* expression (95). Further, when overexpressed, CBP/p300 potentiated CREB, Fos/Jun, C/EBPβ, and GATA-4 transcriptional activity on the *StAR* gene (95, 126). This event is attenuated by the adenovirus E1A oncoprotein, which acts impairing CBP/p300 histone acetyltransferase activity and/or their interaction with other transcription factors or with the basal transcription machinery.

As already reported, coactivators and corepressors also play a key role in the transcriptional regulation of steroid hydroxylase genes. We report here some examples. Coexpression of CBP/p300 with the zinc finger protein TReP-132 has an additive effect on human *CYP11A1* gene promoter activity (163). Similarly, it has been shown that the coactivators CBP/p300, steroid receptor coactivator (SRC)-1, and transcriptional intermediary factor-2 (TIF-2) enhance SF-1-mediated bovine *CYP17* transcription, whereas the corepressors nuclear receptor corepressor (N-CoR) and the silencing mediator of retinoic acid and thyroid hormone receptor (SMRT) increase the repressive activity of chicken ovalbumin upstream-transcription factor 1(COUP-TF-1) (164).

Finally, Sewer and coworkers have shown that a corepressor protein called mSin3A inhibits human *CYP17* gene transcription by the recruitment of a histone deacetylase to the SF-1/NonO/PSF complex that binds to the *CYP17* promoter (58). They have also described that coregulator exchange and sphingosine-sensitive cooperativity of SF-1, general control non-derepressed 5 (GCN5), p54, and p160 coactivators regulate cAMP-dependent *CYP17* transcription rate in H295R cells (165). The same group has shown that ACTH/cAMP signaling pathway promotes acid ceramidase (*ASAH1*) gene transcription via the binding of CREB to multiple region of the *ASAH1* promoter. This event triggers the recruitment of CBP/p300 with a related increase in the trimethylation of Lys4 on histone H3 (H3K4) on the *ASAH1* promoter in H295R cells (166).

## Role of Phosphatase Activity on ACTH/cAMP-Dependent Steroidogenic Gene Transcription

As already discussed, the ACTH-dependent increase in intracellular cAMP levels leads to the activation of PKA, which, in turn, phosphorylates specific nuclear factors to drive steroidogenic gene transcription. Remarkably, in the adrenal cortex ACTH regulation of steroidogenesis depends not only on PKA-mediated Ser/Thr phosphorylation, but also on the activity of protein tyrosine phosphatases (PTPs), which have been implicated in *StAR* expression and steroidogenesis (167–172). Indeed, the phospho-dephosphorylation of intermediate proteins is considered as a key event in the regulation of steroid biosynthesis. In 1999, Paz and coworkers showed that *in vivo* treatment with ACTH leads to an increase in total PTPs activity in adrenal *zona fasciculata*. The stimulation was characterized by a rapid onset (5 min), reached a peak after 15 min of ACTH administration (around twofold) and returned to basal levels after 30 min (168). They showed that the increase in PTPs correlated with a decrease in phosphotyrosine proteins (168). Moreover, the PTPs inhibitors pervanadate (PV) and phenylarsine oxide (PAO) inhibited ACTH- and 8Br-cAMP (a permeant analog of adenosine 3′,5′-phosphate)-dependent steroidogenesis in a dose-dependent fashion, whereas they did not affect steroid production supported by a cell-permeant analog of cholesterol (168). Those studies clearly indicated that PTPs activity has a key role in ACTH/cAMP signaling pathway, acting downstream of PKA activation and upstream of cholesterol transport across the mitochondrial membrane. The same group evaluated steroid production and StAR protein levels in Y1 cells upon PTP inhibition. They reported that PAO reduced ACTH-dependent stimulated steroidogenesis in those cells in a concentration-dependent manner and abrogated StAR protein induction (169). Those effects have been reproduced by a second PTPs inhibitor, benzyl phosphonic acid, which has a different mechanism of action (169). Altogether, those results show that PKA-mediated PTP activation in the steroidogenic system exerts the functional role of mediating StAR protein induction (169). The ACTH/cAMP/PKA signaling pathway stimulates also the release of arachidonic acid (AA) in adrenal and Leydig cells by the concerted action of two enzymes: an acylCoA-thioesterase (Acot2) and an acyl-CoA-synthetase (ACS4) (170, 173). Several reports have shown that AA and its metabolites play a key role in the hormonal control of steroidogenesis by regulating both the expression and function of StAR protein (174, 175). The ACTH/PKA system has been shown to control this pathway upregulating the ACS4 protein levels in adrenal and Leydig cells (175). Two PTP inhibitors both abrogate the ACTH/PKA-dependent ACS4 induction and reduced the effects of cAMP on steroidogenesis and StAR protein levels (175). Interestingly, exogenous AA is able to overcome this PTP-dependent inhibitory effect on StAR protein expression and steroidogenesis (176, 177). Furthermore, Sewer and Waterman have shown that PTP activity is essential for cAMP-dependent transcription of the human *CYP17* gene in H295R cells (178). They also investigated whether the inhibition of PTP activity can impair cAMP-dependent mRNA expression of other steroidogenic genes in the adrenal cortex. They have reported that *CYP11A1*, *CYP11B1/2*, and *CYP21A1* also require PTPs for cAMP-dependent mRNA expression, as the inhibition of both serine/threonine and tyrosine phosphatase activities negatively affected this event (178). Those evidences led those authors to propose a model whereby PKA phosphorylates and activates a dual-specificity phosphatase (DSP), which is able to mediate ACTH/cAMP/PKA-dependent transcription of steroidogenic genes (178, 179). The specific DSP has been identified as mitogen-activated protein phosphatase 1 (MKP-1), whose levels are increased by ACTH and cAMP in H295R cells (180). Moreover, the evidence showing that MKP1 overexpression promotes the transcriptional activity of a human *CYP17* promoter-reporter construct and its silencing decreases cAMP-stimulated *CYP17* gene expression, suggest a role for MKP-1 in cAMP-dependent *CYP17* transcriptional activation (180).

## Summary and Conclusion

The studies outlined here have given an important contribution to the understanding of the impact of ACTH on the regulation of steroidogenic gene expression in the adrenal cortex. Given the pivotal role played by ACTH/cAMP signaling in the acute and chronic regulation of steroid secretion and the implication of those hormones in diverse arrays of fundamental physiological processes, an in-depth investigation is needed to completely elucidate the ACTH-dependent transcriptional regulatory mechanisms that control steroid secretion. Indeed, some aspects addressed in this review still represent a challenge for future studies, which might provide the scientific community with a broader picture of the complex signaling pathways and the intricate transcriptional circuitries that coordinately ensure optimal hormonal output.

## Author Contributions

All authors listed, have made substantial, direct, and intellectual contribution to the work, and approved it for publication.

## Conflict of Interest Statement

The authors declare that the research was conducted in the absence of any commercial or financial relationships that could be construed as a potential conflict of interest.

## References

[1] Simpson ER Waterman MR Regulation of the synthesis of steroidogenic enzymes in adrenal cortical cells by ACTH. Annu Rev Physiol (1988) 50:427–40. 10.1146/annurev.ph.50.030188.002235 2837136

[2] Markowska A Rebuffat P Rocco S Gottardo G Mazzocchi G Nussdorfer GG Evidence that an extrahypothalamic pituitary corticotropin releasing hormone (CRH)/adrenocorticotropin (ACTH) system controls adrenal growth and secretion in rats. Cell Tissue Res (1993) 272:439–45. 10.1007/BF00318550 8393384

[3] Miller WL Bose HS. Early steps in steroidogenesis: intracellular cholesterol trafficking. J Lipid Res (2011) 52:2111–35. 10.1194/jlr.R016675 21976778 PMC3283258

[4] Sewer MB Waterman MR. ACTH modulation of transcription factors responsible for steroid hydroxylase gene expression in the adrenal cortex. Microsc Res Tech (2003) 61:300–7. 10.1002/jemt.10339 12768545

[5] Stocco DM. Tracking the role of a StAR in the sky of the new millennium. Mol Endocrinol (2001) 15:1245–54. 10.1210/mend.15.8.0697 11463850

[6] Manna PR Dyson MT Eubank DW Clark BJ Lalli E Sassone-Corsi P Regulation of steroidogenesis and the steroidogenic acute regulatory protein by a member of the cAMP response-element binding protein family. Mol Endocrinol (2002) 16:184–99. 10.1210/mend.16.1.0759 11773448

[7] Manna PR Eubank DW Lalli E Sassone-Corsi P Stocco DM. Transcriptional regulation of the mouse steroidogenic acute regulatory protein gene by the cAMP response-element binding protein and steroidogenic factor 1. J Mol Endocrinol (2003) 30:381–97. 10.1677/jme.0.0300381 12790807

[8] Manna PR Dyson MT Stocco DM. Regulation of the steroidogenic acute regulatory protein gene expression: present and future perspectives. Mol Hum Reprod (2009) 15:321–33. 10.1093/molehr/gap025 19321517 PMC2676994

[9] Mason JI Rainey WE. Steroidogenesis in the human fetal adrenal: a role for cholesterol synthesized de novo. J Clin Endocrinol Metab (1987) 64:140–7. 10.1210/jcem-64-1-140 3023427

[10] Gwynne JT Strauss JF 3rd The role of lipoproteins in steroidogenesis and cholesterol metabolism in steroidogenic glands. Endocr Rev (1982) 3:299–329. 10.1210/edrv-3-3-299 6288367

[11] Horton JD Goldstein JL Brown MS SREBPs: activators of the complete program of cholesterol and fatty acid synthesis in the liver. J Clin Invest (2002) 109:1125–31. 10.1172/JCI15593 11994399 PMC150968

[12] Brown MS Kovanen PT Goldstein JL Receptor-mediated uptake of lipoprotein-cholesterol and its utilization for steroid synthesis in the adrenal cortex. Recent Prog Horm Res (1979) 35:215–57. 10.1126/science.6261329 229524

[13] Chang TY Chang CC Ohgami N Yamauchi Y. Cholesterol sensing, trafficking and esterification. Annu Rev Cell Dev Biol (2006) 22:129–57. 10.1146/annurev.cellbio.22.010305.104656 16753029

[14] Miller WL Regulation of steroidogenesis by electron transfer. Endocrinology (2005) 146:2544–50. 10.1210/en.2005-0096 15774560

[15] Agarwal AK Auchus RJ Cellular redox state regulates hydroxysteroid dehydrogenase activity and intracellular hormone potency. Endocrinology (2005) 146:2531–8. 10.1210/en.2005-0061 15774561

[16] Penning TM Molecular endocrinology of hydroxysteroid dehydrogenases. Endocr Rev (1997) 18:281–305. 10.1210/edrv.18.3.0302 9183566

[17] Auchus RJ Rainey WE Adrenarche – physiology, biochemistry and human disease. Clin Endocrinol (Oxf) (2004) 60:288–96. 10.1046/j.1365-2265.2003.01858 15008992

[18] Mulatero P Curnow KM Aupetit-Faisant B Foekling M Gomez-Sanchez C Veglio F Recombinant CYP11B genes encode enzymes that can catalyze conversion of 11-deoxycortisol to cortisol, 18-hydroxycortisol, and 18-oxocortisol. J Clin Endocrinol Metab (1998) 83:3996–4001. 10.1210/jcem.83.11.5237 9814482

[19] Kater CE Biglieri EG Disorders of steroid 17α-hydroxylase deficiency. Endocrinol Metab Clin North Am (1994) 23:341–57. 8070426

[20] Voutilainen R Miller WL Coordinate tropic hormone regulation of mRNAs for insulin-like growth factor II and the cholesterol side-chain cleavage enzyme, P450scc, in human steroidogenic tissues. Proc Natl Acad Sci U S A (1987) 84:1590–4. 10.1073/pnas.84.6.1590 3031644 PMC304481

[21] Mesiano S Mellon SH Gospodarowicz D Di Blasio AM Jaffe RB. Basic fibroblast growth factor expression is regulated by corticotropin in the human fetal adrenal: a model for adrenal growth regulation. Proc Natl Acad Sci U S A (1991) 88:5428–32. 10.1073/pnas.88.12.5428 1711231 PMC51886

[22] Coulter CL Read LC Carr BR Tarantal AF Barry S Styne DM. A role for epidermal growth factor in the morphological and functional maturation of the adrenal gland in the fetal rhesus monkey *in vivo*. J Clin Endocrinol Metab (1996) 81:1254–60. 10.1210/jcem.81.3.8772608 8772608

[23] Clark BJ Soo SC Caron KM Ikeda Y Parker KL Stocco DM Hormonal and developmental regulation of the steroidogenic acute regulatory (StAR) protein. Mol Endocrinol (1995) 9:1346–55. 10.1210/mend.9.10.8544843 8544843

[24] Arakane F King SR Du Y Kallen CB Walsh LP Watari H Phosphorylation of steroidogenic acute regulatory protein (StAR) modulates its steroidogenic activity. J Biol Chem (1997) 272:32656–62. 10.1074/jbc.272.51.32656 9405483

[25] Pon LA Hartigan JA Orme-Johnson NR. Acute ACTH regulation of adrenal corticosteroid biosynthesis: rapid accumulation of a phosphoprotein. J Biol Chem (1986) 261:13309–16. 3020029

[26] Epstein LF Orme-Johnson NR. Regulation of steroid hormone biosynthesis: identification of precursors of a phosphoprotein targeted to the mitochondrion in stimulated rat adrenal cortex cells. J Biol Chem (1991) 266:19739–45. 1655794

[27] Clark BJ Wells J King SR Stocco DM. The purification, cloning and expression of a novel luteinizing hormone-induced mitochondrial protein in MA-10 mouse Leydig tumor cells. Characterization of the steroidogenic acute regulatory protein (StAR). J Biol Chem (1994) 269:28314–22. 7961770

[28] Lin D Sugawara T Strauss JF III Clark BJ Stocco DM Saenger P Role of steroidogenic acute regulatory protein in adrenal and gonadal steroidogenesis. Science (1995) 267:1828–31. 10.1126/science.7892608 7892608

[29] Bose HS Sugawara T Strauss JF III Miller WL International Congenital Lipoid Adrenal Hyperplasia Consortium. The pathophysiology and genetics of congenital lipoid adrenal hyperplasia. N Engl J Med (1996) 335:1870–8. 10.1056/NEJM199612193352503 8948562

[30] Caron KM Soo SC Wetsel WC Stocco DM Clark BJ Parker KL. Targeted disruption of the mouse gene encoding steroidogenic acute regulatory protein provides insights into congenital lipoid adrenal hyperplasia. Proc Natl Acad Sci U S A (1997) 94:11540–5. 10.1073/pnas.94.21.11540 9326645 PMC23530

[31] Hasegawa T Zhao L Caron KM Majdic G Suzuki T Shizawa S Developmental roles of the steroidogenic acute regulatory protein (StAR) as revealed by StAR knockout mice. Mol Endocrinol (2000) 14:1462–71. 10.1210/mend.14.9.0515 10976923

[32] Miller WL. Steroidogenic acute regulatory protein (StAR), a novel mitochondrial cholesterol transporter. Biochim Biophys Acta (2007) 1771:663–76. 10.1016/j.bbalip.2007.02.012 17433772

[33] Arakane F Sugawara T Nishino H Liu Z Holt JA Pain D Steroidogenic acute regulatory protein (StAR) retains activity in the absence of its mitochondrial targeting sequence: implications for the mechanism of StAR action. Proc Natl Acad Sci U S A (1996) 93:13731–6. 10.1073/pnas.93.24.13731 8943003 PMC19407

[34] Bose HS Lingappa VR Miller WL. Rapid regulation of steroidogenesis by mitochondrial protein import. Nature (2002) 417:87–91. 10.1038/417087a 11986670

[35] Bose HS Whittal RM Baldwin MA Miller WL. The active form of the steroidogenic acute regulatory protein, StAR, appears to be a molten globule. Proc Natl Acad Sci U S A (1999) 96:7250–5. 10.1073/pnas.96.13.7250 10377400 PMC22068

[36] Baker BY Yaworsky DC Miller WL. A pH-dependent molten globule transition is required for activity of the steroidogenic acute regulatory protein, StAR. J Biol Chem (2005) 280:41753–60. 10.1074/jbc.M510241200 16234239

[37] Tuckey RC Headlam MJ Bose HS Miller WL. Transfer of cholesterol between phospholipid vesicles mediated by the steroidogenic acute regulatory protein (StAR). J Biol Chem (2002) 277:47123–8. 10.1074/jbc.M206965200 12372832

[38] Baker BY Epand RF Epand RM Miller WL Cholesterol binding does not predict activity of the steroidogenic acute regulatory protein, StAR. J Biol Chem (2007) 282:10223–32. 10.1074/jbc.M611221200 17301050

[39] Papadopoulos V Peripheral-type benzodiazepine/diazepam binding inhibitor receptor: biological role in steroidogenic cell function. Endocr Rev (1993) 14:222–40. 10.1210/edrv-14-2-222 8391980

[40] Hauet T Yao ZX Bose HS Wall CT Han Z Li W Peripheral-type benzodiazepine receptor-mediated action of steroidogenic acute regulatory protein on cholesterol entry into Leydig cell mitochondria. Mol Endocrinol (2005) 19:540–54. 10.1210/me.2004-0307 15498831

[41] Bose M Whittal RM Miller WL Bose HS Steroidogenic activity of StAR requires contact with mitochondrial VDAC1 and PCP. J Biol Chem (2008) 283:8837–45. 10.1074/jbc.M709221200 18250166 PMC2276375

[42] Miller WL Strauss JF 3rd. Molecular pathology and mechanism of action of the steroidogenic acute regulatory protein, StAR. J Steroid Biochem Mol Biol (1999) 69:131–41. 10.1016/S0960-0760(98)00153-8 10418987

[43] Christenson LK Johnson PF McAllister JM Strauss JF III. CCAAT/enhancer-binding proteins regulate expression of the human steroidogenic acute regulatory protein (StAR) gene. J Biol Chem (1999) 274:26591–8. 10.1074/jbc.274.37.26591 10473624

[44] Sugawara T Saito M Fujimoto S. Sp1 and SF-1 interact and cooperate in the regulation of human steroidogenic acute regulatory protein gene expression. Endocrinology (2000) 141:2895–903. 10.1210/endo.141.8.7602 10919277

[45] Sugawara T Holt JA Kiriakidou M Strauss JR III. Steroidogenic factor 1-dependent promoter activity of the human steroidogenic acute regulatory protein (StAR) gene. Biochemistry (1996) 35:9052–9. 10.1021/bi960057r 8703908

[46] Manna PR Eubank DW Stocco DM. Assessment of the role of activator protein-1 on transcription of the mouse steroidogenic acute regulatory protein gene. Mol Endocrinol (2004) 18:558–73. 10.1210/me.2003-0223 14673133

[47] Clem BF Hudson EA Clark BJ Cyclic adenosine 3′,5′-monophosphate (cAMP) enhances cAMP-responsive element binding (CREB) protein phosphorylation and phospho-CREB interaction with the mouse steroidogenic acute regulatory protein gene promoter. Endocrinology (2005) 146:1348–56. 10.1210/en.2004-0761 15550512

[48] Hiroi H Christenson LK Chang L Sammel MD Berger SL Strauss JF III. Temporal and spatial changes in transcription factor binding and histone modifications at the steroidogenic acute regulatory protein (StAR) locus associated with StAR transcription. Mol Endocrinol (2004) 18:791–806. 10.1210/me.2003-0305 14726488

[49] Shih MC Chiu YN Hu MC Guo IC Chung BC. Regulation of steroid production: analysis of *Cyp11a1* promoter. Mol Cell Endocrinol (2011) 10:80–4. 10.1016/j.mce.2010.12.017 21195129

[50] Rice DA Kirkman MS Aitken LD Mouw AR Schimmer BP Parker KL. Analysis of the promoter region of the gene encoding mouse cholesterol side-chain cleavage enzyme. J Biol Chem (1990) 15:11713–20. 2365694

[51] Handler JD Schimmer BP Flynn TR Szyf M Seidman JG Parker KL. An enhancer element and a functional cyclic AMP-dependent protein kinase are required for expression of adrenocortical 21-hydroxylase. J Biol Chem (1988) 263:13068–73. 2843506

[52] Wang XL Bassett M Zhang Y Yin S Clyne C White PC Transcriptional regulation of human 11beta-hydroxylase (hCYP11B1). Endocrinology (2000) 141:3587–94. 10.1210/endo.141.10.7689 11014212

[53] Bassett MH Zhang Y White PC Rainey WE. Regulation of human *CYP11B2* and *CYP11B1*: comparing the role of the common CRE/Ad1 element. Endocr Res (2000) 26:941–51. 10.3109/07435800009048620 11196473

[54] Cheng LC Pai TW Li LA. Regulation of human *CYP11B1* and *CYP11B2* promoters by transposable elements and conserved cis elements. Steroids (2012) 77:100–9. 10.1016/j.steroids.2011.10.010 22079243

[55] Rice DA Aitken LD Vandenbark GR Mouw AR Franklin A Schimmer BP A cAMP-responsive element regulates expression of the mouse steroid 11 betahydroxylase gene. J Biol Chem (1989) 264:14011–5. 2547779

[56] Clyne CD Zhang Y Slutsker L Mathis JM White PC Rainey WE. Angiotensin II and potassium regulate human CYP11B2 transcription through common cis-elements. Mol Endocrinol (1997) 11:638–49. 10.1210/mend.11.5.9920 9139807

[57] Waterman MR Biochemical diversity of cAMP-dependent transcription of steroid hydroxylase genes in the adrenal cortex. J Biol Chem (1994) 269:27783–6. 7961700

[58] Sewer MB Nguyen V Huang C Tucker PW Kagawa N Waterman MR Transcriptional activation of human CYP17 in H295R adrenocortical cells depends on complex formation between p54nrb/NonO, PSF and SF-1, a complex which also participates in repression of transcription. Endocrinology (2002) 143:1280–90. 10.1210/endo.143.4.8748 11897684

[59] Lin CJ Martens JWM Miller WL NF-1C, Sp1, and Sp3 are essential for transcription of the human gene for P450c17 (steroid 17a-hydroxylase/17,20 lyase) in human adrenal NCI-H295A cells. Mol Endocrinol (2001) 15:1277–93. 10.1210/mend.15.8.0679 11463853

[60] Lund J Ahlgren R Wu D Kagimoto M Simpson ER Waterman MR Transcriptional regulation of the bovine *CYP17* (P-450_17α_) gene. J Biol Chem (1990) 265:3304–12. 1689300

[61] Bakke M Lund J. Mutually exclusive interactions of two nuclear orphan receptors determine activity of a cyclic adenosine 3′,5′-monophosphate-responsive sequence in the bovine *CYP17* gene. Mol Endocrinol (1995) 9:327–9. 10.1210/mend.9.3.7776979 7776979

[62] Kagawa N Ogo A Takahashi Y Iwamatsu A Waterman M. A cAMP-regulatory sequence (CRS1) of CYP17 is a cellular target for the homeodomain protein Pbx1. J Biol Chem (1994) 269:18716–9. 7913464

[63] Bischof L Kagawa N Moskow JJ Takahashi Y Iwamatsu A Buchberg AM Members of the Meis and Pbx homeodomain proteins families cooperatively bind a cAMP-responsive sequence (CRS1) from bovine *CYP17*. J Biol Chem (1998) 273:7941–8. 10.1074/jbc.273.14.7941 9525891

[64] Kagawa N Waterman MR. Purification and characterization of a transcription factor which appears to regulate cAMP responsiveness of the human *CYP21B* gene. J Biol Chem (1992) 15:25213–9. 1334085

[65] Wijesuriya SD Zhang G Dardis A Miller WL. Transcriptional regulatory elements of the human gene for cytochrome P450c21 (steroid 21-hydroxylase) lie within intron 35 of the linked C4B gene. J Biol Chem (1999) 274:38097–106. 10.1074/jbc.274.53.38097 10608879

[66] Wilson TE Mouw AR Weaver CA Milbrandt J Parker KL. The orphan nuclear receptor NGFI-B regulates expression of the gene encoding steroid 21-hydroxylase. Mol Cell Biol (1993) 13:861–8. 10.1128/MCB.13.2.861 8380897 PMC358969

[67] Parker KL Schimmer BP Chaplin DD Seidman JG. Characterization of a regulatory region of the steroid 21-hydroxylase gene. J Biol Chem (1986) 261:15353–5. 3491069

[68] Schimmer BP Parker KL. Promoter elements of the mouse 21-hydroxylase (Cyp-21) gene involved in cell-selective and cAMP-dependent gene expression. J Steroid Biochem Mol Biol (1992) 43:937–50. 10.1016/0960-0760(92)90322-A 22217839

[69] Milstone DS Shaw SK Parker KL Szyf M Seidman JG. An element regulating adrenal-specific steroid 21-hydroxylase expression is located within the slp gene. J Biol Chem (1992) 267:21924–7. 1400503

[70] Rice DA Mouw AR Bogerd AM Parker KL. A shared promoter element regulates the expression of three steroidogenic enzymes. Mol Endocrinol (1991) 5:1552–61. 10.1210/mend-5-10-1552 1775136

[71] Udhane S Kempna P Hofer G Mullis PE Flück CE. Differential regulation of human 3β-hydroxysteroid dehydrogenase type 2 for steroid hormone biosynthesis by starvation and cyclic AMP stimulation: studies in the human adrenal NCI-H295R cell model. PLoS One (2013) 9:e68691. 10.1371/journal.pone.0068691 23874725 PMC3706324

[72] Lala DS Rice DA Parker KL. Steroidogenic factor I, a key regulator of steroidogenic enzyme expression, is the mouse homolog of fushi tarazu-factor I. Mol Endocrinol (1992) 6:1249–58. 10.1210/mend.6.8.1406703 1406703

[73] Morohashi K Honda S Inomata Y Handa H Omura T. A common trans-acting factor, Ad4-binding protein, to the promoters of steroidogenic P-450s. J Biol Chem (1992) 25:17913–9. 1517227

[74] Hammer GD Krylova I Zhang Y Darimont BD Simpson K Weigel NL Phosphorylation of the nuclear receptor SF-1 modulates cofactor recruitment: integration of hormone signalling in reproduction and stress. Mol Cell (1999) 3:521–6. 10.1016/S1097-2765(00)80480-3 10230405

[75] Ito M Yu RN Jameson JL Steroidogenic factor-I contains a carboxy-terminal transcriptional activation domain that interacts with steroid receptor coactivator-1. Mol Endocrinol (1998) 12:290–301. 10.1210/mend.12.2.0059 9482669

[76] Monte D DeWitte F Hum DW. Regulation of the human P450scc gene by steroidogenic factor 1 is mediated by CBP/p300. J Biol Chem (1998) 273:4585–91. 10.1074/jbc.273.8.4585 9468515

[77] Clark BJ Combs R Angiotensin II and cyclic adenosine 3′,5′-monophosphate induce human steroidogenic acute regulatory protein transcription through a common steroidogenic factor-1 element. Endocrinology (1999) 140:4390–8. 10.1210/endo.140.10.7085 10499490

[78] Caron KM Ikeda Y Soo SC Stocco DM Parker KL Clark BJ. Characterization of the promoter region of the mouse gene encoding the steroidogenic acute regulatory protein. Mol Endocrinol (1997) 11:137–47. 10.1210/mend.11.2.9880 9013761

[79] Sandhoff TW Hales DB Hales KH McLean MP Transcriptional regulation of steroidogenic acute regulatory protein gene by steroidogenic factor 1. Endocrinology (1998) 139:4820–31. 10.1210/endo.139.12.6345 9832418

[80] Hu MC Hsu NC Pai CI Wang CK Chung B. Functions of the upstream and proximal steroidogenic factor 1 (SF-1)-binding sites in the CYP11A1 promoter in basal transcription and hormonal response. Mol Endocrinol (2001) 15:812–28. 10.1210/me.2002-0055 11328860

[81] Takayama K Morohashi K Honda S Hara N Omura T. Contribution of Ad4BP, a steroidogenic cell-specific transcription factor, to regulation of the human CYP11A and bovine CYP11B genes through their distal promoters. J Biochem (1994) 116:193–203. 7798178 10.1093/oxfordjournals.jbchem.a124493

[82] Huang Y Hu M Hsu N Wang CL Chung B. Action of hormone responsive sequence in 2.3 kb promoter of CYP11A1. Mol Cell Endocrinol (2001) 175:205–10. 10.1016/S0303-7207(01)00388-4 11325530

[83] Jimenez P Saner K Mayhew B Rainey WE. GATA-6 is expressed in the human adrenal and regulates transcription of genes required for adrenal androgen biosynthesis. Endocrinology (2003) 144:4285–8. 10.1210/en.2003-0472 12959982

[84] Hiroi N Kino T Bassett M Rainey WE Phung M Abu-Asab M Pituitary homeobox factor 1, a novel transcription factor in the adrenal regulating steroid 11beta-hydroxylase. Horm Metab Res (2003) 35:273–8. 10.1055/s-2003-41301 12915995

[85] Hashimoto T Morohashi K Takayama K Honda S Wada T Handa H Cooperative transcription activation between Ad1, CRE-like element, and other elements in the CYP11B gene promoter. J Biochem (1992) 12:573–5. 1336011 10.1093/oxfordjournals.jbchem.a123941

[86] Morohashi K Zanger UM Honda S Hara M Waterman MR Omura T. Activation of CYP11A and CYP11B gene promoters by the steroidogenic cell-specific transcription factor, Ad4BP. Mol Endocrinol (1993) 7:1196–204. 10.1210/mend.7.9.8247022 8247022

[87] Rodriguez H Hum DW Staels B Miller WL. Transcription of the human genes for cytochrome P450scc and P450c17 is regulated differently in human adrenal NCI-H295 cells than in mouse adrenal Y1 cells. J Clin Endocrinol Metab (1997) 82:365–71. 10.1210/jcem.82.2.3721 9024219

[88] Rice DA Kronenberg MS Mouw AR Aitken LD Franklin A Schimmer BP Multiple regulatory elements determine adrenocortical expression of steroid 21-hydroxylase. J Biol Chem (1990) 265:8052–8. 2335516

[89] Parissenti AM Parker KL Schimmer BP Identification of promoter elements in the mouse 21-hydroxylase (Cyp21) gene that require a functional cyclic adenosine 3′,5′-monophosphate-dependent protein kinase. Mol Endocrinol (1993) 7:283–90. 10.1210/mend.7.2.8385740 8385740

[90] Parker KL Schimmer BP. Transcriptional regulation of the genes encoding the cytochrome P-450 steroid hydroxylases. Vitam Horm (1995) 51:339–70. 10.1016/S0083-6729(08)61044-4 7483327

[91] Meyer TE Habener JF Cyclic adenosine 3′,5′-monophosphate response element binding protein (CREB) and related transcription-activating deoxyribonucleic acid-binding proteins. Endocr Rev (1993) 14:269–90. 10.1210/edrv-14-3-269 8319595

[92] Montminy M. Transcriptional regulation by cyclic AMP. Annu Rev Biochem (1997) 66:807–22. 10.1146/annurev.biochem.66.1.807 9242925

[93] Lalli E Sassone-Corsi P Signal transduction and gene regulation: the nuclear response to cAMP. J Biol Chem (1994) 269:17359–62. 8021233

[94] Sugawara T Holt JA Driscoll D Strauss JF III Lin D Miller WL Human steroidogenic acute regulatory protein: functional activity in COS-1 cells, tissue-specific expression, and mapping of the structural gene to 8p11.2 and a pseudogene to chromosome 13. Proc Natl Acad Sci U S A (1995) 92:4778–82. 10.1073/pnas.92.11.4778 7761400 PMC41790

[95] Silverman E Yivgi-Ohana N Sher N Bell M Eimerl S Orly J. Transcriptional activation of the steroidogenic acute regulatory protein (StAR) gene: GATA-4 and CCAAT/enhancer-binding protein beta confer synergistic responsiveness in hormone-treated rat granulosa and HEK293 cell models. Mol Cell Endocrinol (2006) 252:92–101. 10.1016/j.mce.2006.03.008 16682116

[96] Manna PR Stocco DM. The role of JUN in the regulation of PRKCC-mediated STAR expression and steroidogenesis in mouse Leydig cells. J Mol Endocrinol (2008) 41:329–41. 10.1677/JME-08-0077 18755854

[97] Hummler E Cole TJ Blendy JA Ganss R Aguzzi A Schmid W Targeted mutation of the CREB gene: compensation within the CREB/ATF family of transcription factors. Proc Natl Acad Sci U S A (1994) 91:5647–51. 10.1073/pnas.91.12.5647 8202542 PMC44053

[98] Foulkes NS Sassone-Corsi P More is better: activators and repressors from the same gene. Cell (1992) 68:411–4. 10.1016/0092-8674(92)90178-F 1739963

[99] Meyer TE Habener JF Cyclic AMP response element binding protein CREB and modulator protein CREM are products of distinct genes. Nucleic Acids Res (1992) 20:6106 10.1093/nar/20.22.6106 1461747 PMC334485

[100] Walker WH Habener JF. Role of transcription factors CREB and CREM in cAMP-regulated transcription during spermatogenesis. Trends Endocrinol Metab (1996) 7:133–8. 10.1016/1043-2760(96)00035-5 18406739

[101] Sugawara T Sakuragi N Minakami H. CREM confers cAMP responsiveness in human steroidogenic acute regulatory protein expression in NCI-H295R cells rather than SF-1/Ad4BP. J Endocrinol (2006) 191:327–327. 10.1677/joe.1.06601 17065415

[102] Della Fazia MA Servillo G Sassone-Corsi P. Cyclic AMP signalling and cellular proliferation: regulation of CREB and CREM. FEBS Lett (1997) 410:22–4. 10.1016/S0014-5793(97)00445-6 9247115

[103] Gonzalez GA Montminy MR. Cyclic AMP stimulates somatostatin gene transcription by phosphorylation of CREB at serine 133. Cell (1989) 59:675–80. 10.1016/0092-8674(89)90013-5 2573431

[104] Chrivia JC Kwok RP Lamb N Hagiwara M Montminy MR Goodman RH. Phosphorylated CREB binds specifically to the nuclear protein CBP. Nature (1993) 365:855–9. 10.1038/365855a0 8413673

[105] Parker D Ferreri K Nakajima T LaMorte VJ Evans R Koerber SC Phosphorylation of CREB at Ser-133 induces complex formation with CREB-binding protein via a direct mechanism. Mol Cell Biol (1996) 16:694–703. 10.1128/MCB.16.2.694 8552098 PMC231049

[106] Hiroi H Christenson LK Strauss JF III. Regulation of transcription of the steroidogenic acute regulatory protein (StAR) gene: temporal and spatial changes in transcription factor binding and histone modification. Mol Cell Endocrinol (2004) 215:119–26. 10.1016/j.mce.2003.11.014 15026184

[107] Bonni A Ginty DD Dudek H Greenberg ME. Serine 133-phosphorylated CREB induces transcription via a cooperative mechanism that may confer specificity to neurotrophin signals. Mol Cell Neurosci (1995) 6:168–83. 10.1006/mcne.1995.1015 7551568

[108] Johannessen M Delghandi MP Moens U. What turns CREB on? Cell Signal (2004) 16:1211–27. 10.1016/j.cellsig.2004.05.001 15337521

[109] Struthers RS Vale WW Arias C Sawchenko PE Montminy MR Somatotroph hypoplasia and dwarfism in transgenic mice expressing a nonphosphorylatable CREB mutant. Nature (1991) 350:622–4. 10.1038/350622a0 1826763

[110] Sher N Yivgi-Ohana N Orly J Transcriptional regulation of the cholesterol side chain cleavage cytochrome P450 gene (*CYP11A1*) revisited: binding of GATA, cyclic adenosine 3′,5′-monophosphate response element-binding protein and activating protein (AP)-1 proteins to a distal novel cluster of cis-regulatory elements potentiates AP-2 and steroidogenic Factor-1-dependent gene expression in the rodent placenta and ovary. Mol Endocrinol (2007) 21:948–62. 10.1210/en.2008-054 17213386

[111] Osada S Yamamoto H Nishihara T Imagawa M. DNA binding specificity of the CCAAT/enhancer-binding protein transcription factor family. J Biol Chem (1996) 271:3891–6. 10.1074/jbc.271.7.3891 8632009

[112] Piontkewitz Y Enerback S Hedin L. Expression of CCAAT enhancer binding protein-alpha (C/EBP alpha) in the rat ovary: implications for follicular development and ovulation. Dev Biol (1996) 179:288–96. 10.1006/dbio.1996.0258 8873771

[113] Nalbant D Williams SC Stocco DM Khan SA Luteinizing hormone dependent gene regulation in Leydig cells may be mediated by CCAAT/enhancer-binding protein-beta. Endocrinology (1998) 139:272–9. 10.1210/endo.139.1.5663 9421425

[114] Wilson HL Roesler WJ. CCAAT/enhancer binding proteins: do they possess intrinsic cAMP-inducible activity? Mol Cell Endocrinol (2002) 188:15–20. 10.1016/S0303-7207(01)00754-7 11911941

[115] Reinhart AJ Williams SC Clark BJ Stocco DM SF-1 (steroidogenic factor-1) and C/EBP beta (CCAAT/enhancer binding protein-b) cooperate to regulate the murine StAR (steroidogenic acute regulatory) promoter. Mol Endocrinol (1999) 13:729–41. 10.1210/mend.13.5.0279 10319323

[116] Silverman E Eimerl S Orly J. CCAAT enhancer-binding protein beta and GATA-4 binding regions within the promoter of the steroidogenic acute regulatory protein (StAR) gene are required for transcription in rat ovarian cells. J Biol Chem (1999) 274:17987–96. 10.1074/jbc.274.25.17987 10364248

[117] Tremblay JJ Hamel F Viger RS. Protein kinase A-dependent cooperation between GATA and CCAAT/enhancer-binding protein transcription factors regulates steroidogenic acute regulatory protein promoter activity. Endocrinology (2002) 143:3935–45. 10.1210/en.2002-220413 12239105

[118] Hsu CC Lu CW Huang BM Wu MH Tsai SJ. Cyclic adenosine 3′,5′-monophosphate response element-binding protein and CCAAT/enhancer-binding protein mediate prostaglandin E2-induced steroidogenic acute regulatory protein expression in endometriotic stromal cells. Am J Pathol (2008) 173:433–41. 10.2353/ajpath.2008.080199 18583320 PMC2475780

[119] Sterneck E Tessarollo L Johnson PF. An essential role for C/EBPbeta in female reproduction. Genes Dev (1997) 11:2153–62. 10.1101/gad.11.17.2153 9303532 PMC275394

[120] Shaulian E Karin M AP-1 as a regulator of cell life and death. Nat Cell Biol (2002) 4:131–6. 10.1038/ncb0502-e131 11988758

[121] Sassone-Corsi P Lamph WW Kamps M Verma IM. Fos-associated cellular p39 is related to nuclear transcription factor AP-1. Cell (1988) 54:553–60. 10.1016/0092-8674(88)90077-3 3135941

[122] Curran T Franza BR Jr Fos and Jun: the AP-1 connection. Cell (1998) 55:395–7. 10.1016/0092-8674(88)90024-4 3141060

[123] Halazonetis TD Georgopoulos K Greenberg ME Leder P. c-Jun dimerizes with itself and with c-Fos, forming complexes of different DNA binding affinities. Cell (1988) 55:917–24. 10.1016/0092-8674(88)90147-X 3142692

[124] Hai T Curran T. Cross-family dimerization of transcription factors Fos/Jun and ATF/CREB alters DNA binding specificity. Proc Natl Acad Sci U S A (1991) 1:3720–4. 10.1016/0092-8674(88)90147-X 1827203 PMC51524

[125] Gentz R Rauscher FJ III Abate C Curran T. Parallel association of Fos and Jun leucine zippers juxtaposes DNA binding domains. Science (1989) 243:1695–9. 10.1126/science.2494702 2494702

[126] Manna PR Stocco DM. Crosstalk of CREB and Fos/Jun on a single cis-element: transcriptional repression of the steroidogenic acute regulatory protein gene. J Mol Endocrinol (2007) 39:261–77. 10.1677/JME-07-0065 17909266

[127] Shea-Eaton W Sandhoff TW Lopez D Hales DB McLean MP. Transcriptional repression of the rat steroidogenic acute regulatory (StAR) protein gene by the AP-1 family member c-Fos. Mol Cell Endocrinol (2002) 188:161–70. 10.1016/S0303-7207(01)00715-88 11911955

[128] Lehoux JG Fleury A Ducharme L. The acute and chronic effects of adrenocorticotropin on the levels of messenger ribonucleic acid and protein of steroidogenic enzymes in rat adrenal in vivo. Endocrinology (1998) 139:3913–22. 10.1210/endo.139.9.6196 9724047

[129] Manna PR Chandrala SP Jo Y Stocco DM. cAMP-independent signaling regulates steroidogenesis in mouse Leydig cells in the absence of StAR phosphorylation. J Mol Endocrinol (2006) 37:81–95. 10.1677/jme.1.02065 16901926

[130] Suske G. The Sp-family of transcription factors. Gene (1999) 238:291–300. 10.1016/S0378-1119(99)00357-1 10570957

[131] Kadonaga JT Jones KA Tjian R Promoter-specific activation of RNA polymerase II transcription by Sp1. Trends Biochem Sci (1986) 11:20–3. 10.1016/0968-0004(86)90226-4

[132] Majello B De Luca P Suske G Lania L. Differential transcriptional regulation of c-myc promoter through the same DNA binding sites targeted by Sp1-like proteins. Oncogene (1995) 10:1841–8. 7753559

[133] Yu B Datta PK Bagchi S. Stability of the Sp3-DNA complex is promoter-specific: Sp3 efficiently competes with Sp1 for binding to promoters containing multiple Sp-sites. Nucleic Acids Res (2003) 102:5368–76. 10.1093/nar/gkg706 12954773 PMC203306

[134] Giatzakis C Papadopoulos V. Differential utilization of the promoter of peripheral-type benzodiazepine receptor by steroidogenic versus nonsteroidogenic cell lines and the role of Sp1 and Sp3 in the regulation of basal activity. Endocrinology (2004) 145:1113–23. 10.1210/en.2003-1330 14630713

[135] Chou SJ Lai KN Chung BC. Characterization of the upstream sequence of the human CYP11A1 gene for cell type-specific expression. J Biol Chem (1996) 271:22125–9. 10.1074/jbc.271.36.22125 8703023

[136] Ahlgren R Suske G Waterman MR Lund J. Role of Sp1 in *c*AMP-dependent transcriptional regulation of the bovine CYP11A gene. J Biol Chem (1999) 274:19422–8. 10.1074/jbc.274.27.19422 10383457

[137] Liu Z Simpson ER. Molecular mechanism for cooperation between Sp1 and steroidogenic factor-1 (SF-1) to regulate bovine CYP11A gene expression. Mol Cell Endocrinol (1999) 153:183–96. 10.1016/S0303-7207(99)00036-2 10459866

[138] Urban RJ Bodenburg Y Kurosky A Wood TG Gasic S. Polypyrimidine tract-binding protein-associated splicing factor is a negative regulator of transcriptional activity of the porcine P450scc insulin-like growth factor response element. Mol Endocrinol (2000) 14:774–82. 10.1210/mend.14.6.0485 10847580

[139] Fluck CE Miller WL. GATA-4 and GATA-6 modulate tissue-specific transcription of the human gene for P450c17 by direct interaction with Sp1. Mol Endocrinol (2004) 18:1144–57. 10.1210/me.2003-0342 14988427

[140] Zanger UM Kagawa N Lund J Waterman MR. Distinct biochemical mechanisms for cAMP-dependent transcription of CYP17 and CYP21. FASEB J (1992) 6:719–23. 1311271 10.1096/fasebj.6.2.1311271

[141] Zanaria E Muscatelli F Bardoni B Strom TM Guioli S Guo W An unusual member of the nuclear hormone receptor superfamily responsible for X-linked adrenal hypoplasia congenita. Nature (1994) 372:635–41. 10.1038/372635a0 7990953

[142] Muscatelli F Strom TM Walker AP Zanaria E Récan D Meindl A Mutations in the DAX-1 gene give rise to both X-linked adrenal hypoplasia congenita and hypogonadotropic hypogonadism. Nature (1994) 372:672–6. 10.1038/372672a0 7990958

[143] Zazopoulos E Lalli E Stocco DM Sassone-Corsi P. DNA binding and transcriptional repression by DAX-1 blocks steroidogenesis. Nature (1997) 390:311–5. 10.1038/36899 9384387

[144] Ito M Yu R Jameson JL. DAX-1 inhibits SF-1-mediated transactivation via a carboxy-terminal domain that is deleted in adrenal hypoplasia congenita. Mol Cell Biol (1997) 17:1476–83. 10.1128/MCB.17.3.1476 9032275 PMC231873

[145] Lalli E Melner MH Stocco DM Sassone-Corsi P. DAX-1 blocks steroid production at multiple levels. Endocrinology (1998) 139:4237–43. 10.1210/en.139.10.4237 9751505

[146] Lalli E Role of orphan nuclear receptor DAX-1 in development, physiology and disease. Adv Biol (2014). 10.1155/2014/582749

[147] Ragazzon B Lefrançois-Martinez AM Val P Sahut-Barnola I Tournaire C Chambon C Adrenocorticotropin-dependent changes in SF-1/DAX-1 ratio influence steroidogenic genes expression in a novel model of glucocorticoid-producing adrenocortical cell lines derived from targeted tumorigenesis. Endocrinology (2006) 147:1805–18. 10.1210/en.2005-1279 16439455

[148] Tamai KT Monaco L Alastalo TP Lalli E Parvinen M Sassone-Corsi P. Hormonal and developmental regulation of DAX-1 expression in Sertoli cells. Mol Endocrinol (1996) 10:1561–9. 10.1210/me.10.12.1561 8961266

[149] McKenna NJ O’Malley BW Nuclear receptor coregulators. Pure Appl Chem (2003) 75:1665–9. 10.1351/pac200375111665

[150] McKenna NJ Xu J Nawaz Z Tsai SY Tsai M-J O’Malley BW. Nuclear receptor coactivators: multiple enzymes, multiple complexes, multiple functions. J Steroid Biochem Mol Biol (1999) 69:3–12. 10.1016/S0960-0760(98)00144-7 10418975

[151] Lonard DM O’Malley BW. Expanding functional diversity of the coactivators. Trends Biochem Sci (2005) 30:126–32. 10.1016/j.tibs.2005.01.001 15752984

[152] Spencer TE Jenster G Burcin MM Allis CD Zhou J Mizzen CA Steroid receptor coactivator-1 is a histone acetyltransferase. Nature (1997) 389:194–8. 10.1038/38304 9296499

[153] Chen H Lin RJ Schiltz RL Chakravarti D Nash A Nagy L Nuclear receptor coactivator ACTR is a novel histone acetyltransferase and forms a multimeric activation complex with P/CAF and CBP/p300. Cell (1997) 90:569–80. 10.1016/S0092-8674(00)80516-4 9267036

[154] Vo N Goodman RH CREB-binding protein and p300 in transcriptional regulation. J Biol Chem (2001) 276:13505–8. 10.1074/jbc.R000025200 11279224

[155] Chen J Kinyamu K Archer TK. Changes in attitude, changes in latitude: nuclear receptors remodeling chromatin to regulate transcription. Mol Endocrinol (2006) 20:1–13. 10.1210/me.2005-0192 16002433

[156] Chen H Lin R Xie W Wilpitz D Evans R. Regulation of hormone-induced histone hyperacetylation and gene activation via acetylation of an acetylase. Cell (1999) 98:675–86. 10.1016/S0092-8674(00)80054-9 10490106

[157] Chen WY Juan LJ Chung BC. SF-1 (nuclear receptor 5A1) activity is activated by cyclic AMP via p300-mediated recruitment to active foci, acetylation, and increased DNA binding. Mol Cell Biol (2005) 25:10442–53. 10.1128/MCB.25.23.10442-10453.2005 16287857 PMC1291237

[158] An W Kim J Roeder RG. Ordered cooperative functions of PRMT1, p300, and CARM1 in transcriptional activation by p53. Cell (2004) 117:735–48. 10.1016/j.cell.2004.05.009 15186775

[159] Perissi V Rosenfeld MG. Controlling nuclear receptors: the circular logic of cofactor cycles. Nat Rev Mol Cell Biol (2005) 6:542–54. 10.1038/nrm1682 15957004

[160] Bannister AJ Kouzarides T. The CBP co-activator is a histone acetyltransferase. Nature (1996) 384:641–3. 10.1038/384641a0 8967953

[161] Fronsdal K Engedal N Slagsvold T Saatcioglu F. CREB binding protein is a coactivator for the androgen receptor and mediates cross-talk with AP-1. J Biol Chem (1998) 273:31853–9. 10.1074/jbc.273.48.31853 9822653

[162] Kovacs KA Steinmann M Magistretti PJ Halfon O Cardinaux JR CCAAT/enhancer-binding protein family members recruit the coactivator CREBbinding protein and trigger its phosphorylation. J Biol Chem (2003) 278:36959–65. 10.1074/jbc.M303147200 12857754

[163] Gizard F Lavallee B DeWitte F Hum DW. A novel zinc finger protein TReP-132 interacts with CBP/p300 to regulate human CYP11A1 gene expression. J Biol Chem (2001) 276:33881–92. 10.1074/jbc.M100113200 11349124

[164] Shibata H Ando T Kurihara I Suzuki T Lund J Morohashi K Functional role of COUP-TF1, SF-1, and nuclear receptor coregulators in the steroidogenesis of adrenocortical adenomas. In: Nawata H, editor. Molecular Steroidogenesis. Okamoto MIY. Tokyo: J Universal Academy Press (2000). p. 345–8.

[165] Dammer EB Leon A Sewer MB. Coregulator exchange and sphingosine-sensitive cooperativity of steroidogenic factor-1, general control nonderepressed 5, p54, and p160 coactivators regulate cyclic adenosine 3′,5′-monophosphate-dependent cytochrome P450c17 transcription rate. Mol Endocrinol (2007) 21:415–38. 10.1210/me.2006-0361 17121866

[166] Lucki N Sewer M. The cAMP-responsive element binding protein (CREB) regulates the expression of acid ceramidase (ASAH1) in H295R human adrenocortical cells. Biochim Biophys Acta (2009) 1791:706–13. 10.1016/j.bbalip.2009.03.005 19298866 PMC3976422

[167] Sayed SB Jones PM Persaud SJ Whitehouse BJ Effects of phosphoprotein phosphatase inhibitors on steroidogenesis and StAR protein expression in Y1 cells. Endocr Res (1998) 24:413–4. 10.3109/07435809809032624 9888516

[168] Paz C Cornejo MacIel F Mendez C Podesta EJ. Corticotropin increases protein tyrosine phosphatase activity by a cAMP-dependent mechanism in rat adrenal gland. Eur J Biochem (1999) 265:911–8. 10.1046/j.1432-1327.1999.00759.x 10518784

[169] Poderoso C Cornejo Maciel F Gorostizaga A Bey P Paz C Podestá EJ. The obligatory action of protein tyrosine phosphatases in ACTH-stimulated steroidogenesis is exerted at the level of StAR protein. Endocr Res (2002) 28:413–7. 10.1081/ERC-120016816 12530643

[170] Castillo F Cano F Maloberti P Castilla R Neuman I Poderoso C Tyrosine phosphates act on steroidogenesis through the activation of arachidonic acid release. Endocr Res (2004) 30:623–7. 10.1081/ERC-200043795 15666802

[171] Gorostizaga A Cornejo Maciel F Brion L Maloberti P Podestá EJ Paz C. Tyrosine phosphatases in steroidogenic cells: regulation and function. Mol Cell Endocrinol (2007) 26(5–266):131–7. 10.1016/j.mce.2006.12.009 17207923

[172] Cooke M Mele P Maloberti P Duarte A Poderoso C Orlando U Tyrosine phosphatases as key regulators of StAR induction and cholesterol transport: SHP2 as a potential tyrosine phosphatase involved in steroid synthesis. Mol Cell Endocrinol (2011) 336:63–9. 10.1016/j.mce.2010.11.030 21145937

[173] Maloberti P Castilla R Castillo F Cornejo Maciel F Mendez CF Paz C Silencing the expression of mitochondrial acyl-CoA thioesterase I and acyl-CoA synthetase 4 inhibits hormone-induced steroidogenesis. FEBS J (2005) 272:1804–14. 10.1111/j.1742-4658.2005.04616.x 15794766

[174] Mele PG Dada LA Paz C Cymeryng CB Cornejo Maciel MF Neuman MI Site of action of proteinases in the activation of steroidogenesis in rat adrenal gland. Biochim Biophys Acta (1996) 29:260–8. 10.1016/0167-4889(95)00177-8 8599603

[175] Wang XJ Dyson MT Jo Y Eubank DW Stocco DM. Involvement of 5-lipoxygenase metabolites of arachidonic acid in cyclic AMP-stimulated steroidogenesis and steroidogenic acute regulatory protein gene expression. J Steroid Biochem Mol Biol (2003) 85:159–66. 10.1016/S0960-0760(03)00189-4 12943700

[176] Cornejo Maciel F Maloberti P Neuman I Cano F Castilla R Castillo F An arachidonic acid-preferring acyl-CoA synthetase is a hormone-dependent and obligatory protein in the signal transduction pathway of steroidogenic hormones. J Mol Endocrinol (2005) 3:655–66. 10.1677/jme.1.01691 15956337

[177] Cano F Poderoso C Cornejo Maciel F Castilla R Maloberti P Castillo F Protein tyrosine phosphatases regulate arachidonic acid release, StAR induction and steroidogenesis acting on a hormone-dependent arachidonic acid-preferring acyl-CoA synthetase. J Steroid Biochem Mol Biol (2006) 99:197–202. 10.1016/j.jsbmb.2006.01.003 16630718

[178] Sewer MB Waterman MR. Adrenocorticotropin/cyclic adenosine 3′,5′-monophosphate-mediated transcription of the human CYP17 gene in the adrenal cortex is dependent on phosphatase activity. Endocrinology (2002) 143:1769–77. 10.1210/endo.143.5.8820 11956159

[179] Sewer MB Waterman MR. cAMP-dependent transcription of steroidogenic genes in the human adrenal cortex requires a dual-specificity phosphatase in addition to protein kinase A. J Mol Endocrinol (2002) 29:163–74. 10.1677/jme.0.0290163 12200237

[180] Sewer MB Waterman MR. cAMP-dependent protein kinase enhances CYP17 transcription via MKP-1 activation in H295R human adrenocortical cells. J Biol Chem (2003) 7:8106–11. 10.1074/jbc.M210264200 12506119

